# Ischemic stroke in COVID-19 patients: a cross-sectional study from an Indonesian COVID-19 referral hospital

**DOI:** 10.1186/s41983-022-00528-z

**Published:** 2022-08-17

**Authors:** Rakhmad Hidayat, Nita Widjaya, Zlatikha Djuliannisaa, Alyssa Putri Mustika, Ramdinal Aviesena Zairinal, Dinda Diafiri, Taufik Mesiano, Mohammad Kurniawan, Al Rasyid, Salim Harris

**Affiliations:** 1 Faculty of Medicine University of Indonesia, Depok, Indonesia; 2 Dr. Cipto Mangunkusumo Hospital, Jakarta, Indonesia; 3 University of Indonesia Hospital, Depok, Indonesia

**Keywords:** COVID-19, Ischemic stroke, Stroke

## Abstract

**Background:**

COVID-19 increases the risk of acute ischemic stroke. The objective of this study is to identify the risk factors, comorbidities, and outcomes in COVID-19 patients with acute ischemic stroke at University of Indonesia Hospital.

**Results:**

The clinical condition of COVID-19 patients with ischemic stroke is more severe for patients older than 55 years (*p* < 0.05), patients at the critical COVID-19 clinical stage, and patients with atrial fibrillation (*p* < 0.05). The level of C-reactive protein (CRP) more than 60 mg/L correlated with the outcome of the patient as well.

**Conclusion:**

The study concluded that, age, COVID-19 clinical degree, and atrial fibrillation significantly affect the outcome in COVID-19 patients with stroke.

## Background

Corona virus disease 2019 (COVID-19) infection is a dangerous infection because of its ability to induce multiorgan failure in humans. Aside from the respiratory system, it may affect the cerebrovascular system, which is manifested as a stroke that occurred in that patient. Stroke is one of the neurological diseases that have high morbidity and mortality. Moreover, treating this condition creates a high economic burden [1]. The World Health Organization (WHO) reported that the risk of ischemic stroke in COVID-19 patients is approximately 5% (95% confidence interval [CI] 2.8–8.7] with a mortality rate 3.2–7.8 times higher (about 38%) than the stroke patients without COVID-19 [2].

The COVID-19 infection increases the risk of ischemic stroke occurrence by several mechanisms, including, activation of coagulation and inflammatory factors reflected on the increased d-dimer level, erythrocyte sedimentation rate, and lactic acid dehydrogenase enzyme, and the decrease in lymphocyte. In addition to, the elevation of the liver enzyme and kidney biomarkers which suspected to play roles in those patients [3].

The severity of COVID-19 infection detected clinically based on the guidelines of the Decree of the Minister of Health of the Republic of Indonesia Number Hk.01.07/Menkes/4641/2021 concerning the Control of COVID-19. The mild degree of the COVID-19 is symptomatic without dyspnea, and the severe-critical degree is acute respiratory distress syndrome (ARDS) with oxygen saturation < 93%, sepsis and septic shock [4].

In Indonesia, University of Indonesia Hospital had reported the cases of stroke in COVID-19 patients without vascular comorbid. However, a greater amount of data was required to understand better the risk factors in COVID-19 that increased the risk of ischemic stroke. Finally, understanding the pathophysiology of stroke in COVID-19 patients and the mechanisms of each risk factor was essential as consideration for COVID-19 therapies and the prognosis of stroke in COVID-19 patients.

## Method

This study was a cross-sectional study where patients’ electronic medical records were tracked from March 2020 to August 2021. The inclusion criteria for these patients were the hospitalized patients with positive COVID-19 infection identified by real-time PCR tests that stated the clinical symptoms of stroke and were supported by brain CT scans and MRI procedures. There were 52 stroke patients selected from a total of 26,154 hospitalized COVID-19 patients. The risk factors of the patients which were analyzed include age, gender, stroke types, hypertension, diabetes, hyperlipidemia, atrial fibrillation, chronic kidney disease, coronary artery disease, thorax radiology, COVID-19 severity degree, inflammatory markers such as d-dimer, C-reactive protein (CRP), and procalcitonin. D-dimer was measured by using FREND System manufactured in South Korea by NanoEntek company while CRP test was carried out by using Epithod 616 machine that was manufactured in South Korea by DxGen Corp. Procalcitonin test was measured by RAMP System manufactured in Canada by Response Medical Corp. The collected data were analyzed using the SPSS-26 application produced by IBM in Chicago in April 2019 and by comparing risk factors and the comorbidities of patients we may identify the dominant factors which play more roles in causing the stroke.

## Results

The majority of the patients (82.7%) were older than 55 years old. Male numbers (61.5%) dominated the number of female patients (38.5%). The ischemic stroke was reported in 96.2% of patients, and hypertension (69.2%) was the most frequent risk factor followed by atrial fibrillation (19.2%). Most of the patients (98.1%, and 98%, respectively) demonstrated an elevation in d-dimer and CRP, while procalcitonin level increased in about half of the patients (51.1%) (Table 1).

Out of 52 patients, there were 49 patients under cover of anticoagulant drugs as, 43 patients were treated with heparin (82.7%), five patients (9.6%) were treated with LMWH, and only one patient (1.9%) under oral anticoagulant (Table 1).

**Table 1:** Demographic characteristics and the risk factors of the patients

	Number	Percentage %
Age (*n* = 52)		
< 55 years old	9	17.3
> 55 years old	43	82.7
Gender (*n* = 52)		
Male	32	61.5
Female	20	38.5
Stroke type (*n* = 52)		
Ischemic	50	96.2
Hemorrhagic	2	3.8
Hypertension (*n* = 52)		
Yes	36	69.2
No	16	30.8
Diabetes (*n* = 52)		
Yes	23	44.2
No	29	55.8
Hyperlipidemia (*n* = 52)		
Yes	18	34.6
No	34	65.4
Atrial fibrillation (*n* = 52)		
Yes	10	19.2
No	42	80.8
CKD (*n* = 52)		
Yes	25	48.1
No	27	51.9
CAD (*n* = 52)		
Yes	11	21.2
No	41	78.8
MRS (*n* = 52)		
Score 0–2	11	21.2
Score 3–5	10	19.2
Score 6	31	59.6
d-dimer (*n* = 52)		
Normal	1	1.9
Increasing	51	98.1
CRP (*n* = 51)		
Normal	1	2
Increasing	50	98
Procalcitonin (*n* = 47)		
Normal	23	48.9
Increasing	24	51.1
Patients with anticoagulant (*n* = 49)		
Oral	1	1.9
Heparin	43	82.7
LMWH	5	9.6

*CKD* chronic kidney disease, *CAD* coronary artery disease, *MRS* Modified Rankin Scale, *LMWH* low-molecular heparin

The results revealed that the age average among 52 patients was 66 years (95% confidence interval [CI] 63.04–69.27). The systolic and diastolic pressure averages were 140.50 mmHg and 79.58 mmHg, respectively. The MAP was 100.85 mmHg. The leukocyte count average was 14.07 × 103/µL, lymphocyte was 14.07, and neutrophil was 80.84%. The erythrocyte count sedimentation average rate was 58.75 mm. The urea average level was 87.15 mg/dL. Then, the creatinine level was 2.03. The SGOT and SGPT level were 70.46 U/L and 49.61 U/L, respectively. Next, the HbA1C was 6.43. The D-dimer and CRP were 9.93 mg/L and 101.31 mg/L, respectively. Finally, the procalcitonin was 4.25 ng/mL (Table 2).

**Table 2:** The clinical, laboratory data of the patients and the 95% confidence interval

	*N*	Minimum	Maximum	Average	CI 95%
Age (years old)	52	38	89	66.14	63.04–69.27
Systolic (mmHg)	52	97	224	140.5	133.71–147.86
Diastolic (mmHg)	52	54	105	79.58	75.75–83.19
MAP (mmHg)	52	70	138	100.85	96.58–105.10
Leukocyte (g/dL)	52	3.22	65.98	14.07	11.46–17.45
Lymphocyte (%)	52	0.70	33	9.85	6.06–14.15
Neutrophil (%)	52	48.10	96.30	80.84	74.25–86.64
ESR (mm)	16	6	142	58.75	41.75–77.68
APTT (s)	50	18.80	190	38.73	31.45–48.12
PT (s)	48	9	17.90	10.99	10.58–11.47
Urea (mg/dL)	52	14.00	346.00	87.15	67.73–109.24
Creatinine (mg/dL)	52	0.28	11.54	2.03	1.47–2.71
eGFR (mL/min/1.73 m^2^)	52	4.20	148.40	57.36	48.43–68.45
SGOT (IU/L)	52	11.00	631.00	70.46	51.25–98.71
SGPT (IU/L)	52	4	411.00	49.61	36.06–69.80
Total cholesterol (mg/dL)	9	100	214	150.44	128.22–173.66
Triglycerides (mg/dL)	10	46.00	155.00	102.60	84.50–120.89
LDL (mg/dL)	10	59.00	246.00	106.10	81.-141.59
HbA1C (%)	23	4.20	10.60	6.43	5.73–7.23
d-dimer (ng/mL)	52	0.19	9.93	2.93	2.32–3.57
CRP (mg/L)	51	4.00	236	101.31	84.24–119.08
Procalcitonin (ng/mL)	47	0.02	66.00	4.25	1.81–7.81

*MAP* mean arterial pressure, *ESR* erythrocyte sedimentation rate, *APTT* activated partial thromboplastin time, *PT* prothrombin time, *eGFR* estimated glomerular filtration rate, *SGOT* serum glutamic oxaloacetic transaminase, *SGPT* serum glutamic pyruvic transaminase, *LDL* low-density lipoprotein, *HbA1C* hemoglobin A1c, *CRP* C-reactive protein

There were 43 out of 52 patients older than 55 years (Table 3). Out of 43 people, 29 passed away. The researcher found that there were 10 out of 52 patients had atrial fibrillation, 9 of whom eventually passed away. The limitation for CRP level in this study was 60 mg/L. There were 51 patients whose CRP levels were higher than 60 mg/L, 22 of whom eventually passed away. Procalcitonin point was 0.2 ng/. Out of 47 patients, 41 had high procalcitonin and 28 passed away. In this study, the researcher divided the patients into two groups based on the COVID-19 severity. The researcher discovered 39 out of 52 patients with critical COVID-19 conditions and 30 of them passed away.

**Table 3:** Risk factors and outcome

	Alive	Deceased	*p*-value	OR	Cl 95%
Age (years old)			0.022	7.250	1.33–39.52
< 55	7	2			
> 55	14	29			
Gender			1.000	1.026	0.328–3.207
Male	13	19			
Female	8	12			
Stroke type			1.000	0.667	0.39–11.285
Ischemic	20	30			
Hemorrhagic	1	1			
Hypertension			1.000	1.190	0.355–3.991
Yes	15	21			
No	6	10			
Diabetes			1.000	0.911	0.298–2.782
Yes	9	14			
No	12	17			
Hyperlipidemia			0.378	1.833	0.574–5.855
Yes	9	9			
No	12	22			
Atrial fibrillation			0.036	0.122	0.014–1.052
Yes	1	9			
No	20	22			
CKD			0.97	0.361	0.114–1.145
Yes	7	18			
No	14	13			
CAD			0.491	0.479	0.111–2.070
Yes	3	8			
No	18	23			
d-dimer (ng/mL)			0.575	0.692	0.218–2.196
< 1700	7	13			
> 1700	14	18			
CRP (mg/L)			0.082	3.025	0.931–9.827
< 60	11	8			
> 60	10	22			
Procalcitonin (ng/mL)					
< 0.2	6	0	0.003		
> 0.2	13	28			
COVID-19 severity			0.000	4.000	4.559–350.942
Mild	12	1			
Critical	9	30			
Bamford score			0.721	0.615	0.154–2.464
LACS	16	26			
Non-LACS	5	5			

*CKD* chronic kidney disease, *CAD* coronary artery disease, *CRP* C-reactive protein, *COVID-19* Coronavirus disease 2019, *LACS* lacunar stroke

## Discussion

In line with the other studies [5, 6], we found that ischemic stroke was the commonest type in this study (96.2%). Mathew and his colleagues [5], reported that 97% of patients suffered from ischemic stroke and merely 3% suffered from hemorrhagic stroke [5]. Nannoni and his colleagues [6] also demonstrated relatively similar results as out of 108,571 COVID-19 patients, about 1.4% of them suffered from stroke, 87.4% experienced ischemic stroke and only 11.6% experienced hemorrhagic type [6]. The sole reason of ischemic stroke pathophysiology was the hypercoagulation that occurred in COVID-19 patients. The hypercoagulation state caused by increased inflammation response, including coagulopathy, indicated the high d-dimer level, as demonstrated in this study. The thrombosis also corresponded to the high level of antibodies and antiphospholipid discovered in COVID-19 patients as well [7].

The patients age limit in this study was 55 years which is in line with recent study which indicated that stroke was discovered in patients aged older than 55 years old [1]. This study reported that patients older than 55 years had a significantly worse outcome than patients younger than 55 years. This result was supported by a study conducted by Fridman and his colleagues [8] which found that patients younger than 50 years have a lesser mortality rate compared to patients older than 70 years old [8]. Yanez and his colleagues [9] found that the incidence rate ratio (IRR) of patients 54 years or under was 8.1, indicating that the mortality rate of COVID-19 was 8.1 times higher (95% CI 7.7, 8.5) among patients age between 55 and 64 years, and more than 62 times higher (IRR = 62.1; 95% CI 59.7, 64.7) among those 65 and older [9]. Qureshi and his colleagues [10] also mentioned that in their study, one of the risk factors related to higher mortality in stroke patients with COVID-19 was older age. There were more patients older than 35 years who passed away compared to those younger than 35 years [10]. One of the most possible explanations may be due to the higher chance among older patients to be infected with a more severe type of COVID-19, so that, the outcome was more worse in this group.

This study illustrated that patients with ischemic stroke were prone to have a worse outcome and higher mortality rate compared to patients with hemorrhagic stroke which contradicts a previous cohort study carried out by Syahruland his colleagues [10], where the mortality rate of hemorrhagic stroke patients with COVID-19 was 44.72%, while for the ischemic, 36.23% [11]. This might be due to the small number of patients the study’s sample played a big role as, even though the incidence of hemorrhagic stroke in COVID-19 patients was relatively low. The high mortality rate of ischemic stroke in COVID-19 patients was also demonstrated in a study carried out by Harrison and his colleagues [12] in which 954 patients with ischemic stroke and COVID-19 infection had a higher mortality rate at 60 days compared to non-COVID-19 ischemic stroke patients [12]. This is possibly due to multiorgan failures that occur in COVID-19 patients, therefore the morbidity and mortality chance significantly increased.

Atrial fibrillation was another condition that worsened the outcome of stroke patients with COVID-19 infection. A study conducted by Qureshi and his colleagues [10] indicated that the possibility for COVID-19 patients to experience atrial fibrillation was lower compared to non-COVID-19 patients [10]. That supported this study findings, where only 19.2% of stroke patients with COVID-19 suffered from atrial fibrillation. Furthermore, the patients with atrial fibrillation had a significantly worse outcome and with higher mortality compared to those without such condition (*p* = 0.036). This could be due to the fact that atrial fibrillation was one of the independent ischemic stroke risk factors [13].

This study also reported that the more severe clinical conditions of COVID-19 infection significantly affected the outcome of stroke patients with the higher mortality rate compared to stroke patients with moderate COVID-19 infection (*p*: 0.00) as severe or critical COVID-19 conditions increased the patients' mortality by 40 times (OR: 40, *p*: 0.00, 95% CI 4.5–350). This is supported by a study conducted by Vidale and his colleagues [14] that the severity of stroke was significantly in line with the severity of COVID-19 infection [14]. Fridman and his colleagues [8] as well discovered that the mortality risk was three times higher compared to other severity levels of COVID-19 infection [8].

Based on a study carried out by Lodigiani and his colleagues [15], the median of d-dimer level for survived patients was 353 ng/mL (μg/L), which increased to 529 ng/mL after 1 week compared to patients who passed away with a higher initial point of d-dimer at 869 ng/mL and 1494 ng/mL at the end of the week [15]. In this study, 98.1% of patients administered an increase in d-dimer, however, a significant cutoff was not identified. This finding differed from the study carried out by Tang and his colleagues [16], which indicated that an increased d-dimer level in COVID-19 patients was closely responsible for poor prognosis and higher mortality rate [16]. This could be due to a lack of samples for better understanding the relationship between an increase in d-dimer level and mortality outcome. The high d-dimer was suspected due to the inflammation in COVID-19 infection, which leads to the coagulation cascade. Therefore, the significance of mortality etiology was still inconclusive.

C-reactive protein or CRP serum was discovered to be an essential marker, which might change significantly in severe COVID-19 patients. CRP was a protein produced by the liver, which had a role as an initial marker of infection and inflammation. In this study, the elevated CRP was observed in 86% of severe COVID-19 patients and more than 60 mg/L of CRP was reported as a cut-off point and thrice tendency for higher mortality (OR: 3.025, 95% CI 0.931–9.827) (*p*: 0,08). This could be an important finding which differed from other studies considering a meta-analysis study conducted by Yassin and his colleagues [17] which demonstrated that CRP level was not different between stroke patients with COVID-19 and without COVID-19 infection [17].

Fridman and his colleagues [8] stated that comorbidity plays an important role in determining the mortality of stroke patients with COVID-19 infection. This was in line with a finding which stated that hyperlipidemia patients were inclined to a higher mortality rate (*p*: 1.833, 95% CI 0.574–5.855) [8]. Meanwhile, for the other comorbidities, such as diabetes, this study indicated that there was no significant correlation between diabetes and stroke in COVID-19 patients and the mortality rate. In a study carried out by Jillella and his colleagues [18], it was concluded that, out of 13 stroke patients with COVID-19, 9 patients had diabetes with two of them were being in a prediabetic state [18]. Meanwhile, in a study conducted by Qureshi and his colleagues [10], 58 out of 103 (56.3%) stroke patients with COVID-19 infection, had diabetes, while there was 51.8% of non-COVID-19 stroke patients who had diabetes. Other comorbidities, such as hypertension, CKD, and CAD, did not demonstrate significant results in determining mortality in this study.

## Conclusion

Age of the patients significantly affected the outcome in stroke patients with COVID-19, where the older the patients age, atrial fibrillation, and the more severe the COVID-19 infection, associated with worse outcome, and the higher the chance of mortality. The study also suggested that CRP of higher than 60 mg/L played an important role. Further studies with a higher sample number are required to better understand the pathophysiology behind these findings.

## Data Availability

The datasets generated and/or analyzed during the current study are not publicly available due to patients’ privacy concerns, but are available from the corresponding author on reasonable request.

## Abbreviations

COVID-19: Coronavirus disease 2019
PCR: Polymerase chain reaction
CT scan: Computerized tomography scan
MRI: Magnetic resonance imaging
CRP: C-reactive protein
CKD: Chronic kidney disease
CAD: Coronary artery disease
MRS: Modified Rankin Scale
LMWH: Low-molecular-weight heparin
CI: Confidence interval
MAP: Mean arterial pressure
ESR: Erythrocyte sedimentation rate
APTT: Activated partial thromboplastin time
PT: Prothrombin time
eGFR: Estimated glomerular filtration rate
SGOT: Serum glutamic oxaloacetic transaminase
SGPT: Serum glutamic pyruvic transaminase
LDL: Low-density lipoprotein
OR: Odds ratio
HbA1C: Hemoglobin A1c
LACS: Lacunar stroke
